# Linking five-factor personality model to alcohol consumption in adolescence: a systematic review and meta-analysis

**DOI:** 10.1093/alcalc/agag048

**Published:** 2026-08-10

**Authors:** Singal Aakriti, Priscilla Adasi, Ellen Gustafsson, Sofia Therén-Waern, Johan Melander Hagborg, Ann-Sophie Lindqvist Bagge

**Affiliations:** Department of Psychology, University of Gothenburg, s-405 30 Gothenburg, Sweden; Department of Psychology, University of Gothenburg, s-405 30 Gothenburg, Sweden; Department of Psychology, University of Gothenburg, s-405 30 Gothenburg, Sweden; Department of Psychology, University of Gothenburg, s-405 30 Gothenburg, Sweden; Department of Psychology, University of Gothenburg, s-405 30 Gothenburg, Sweden; Department of Psychology, University of Gothenburg, s-405 30 Gothenburg, Sweden

**Keywords:** alcohol consumption, adolescence, big five, Five Factor Model, personality trait

## Abstract

**Aims:**

This meta-analysis synthesizes empirical research on the association between alcohol consumption, operationalized as both volume and frequency, and Five Factor Model of Personality Traits.

**Methods:**

A systematic literature search was conducted on June 18, 2025, in PsycINFO and PubMed. The search strategy employed the following terms: tiab(“big 5” OR “big five” OR “5 factor” OR “five factor”) AND tiab(alcohol). Studies meeting predefined inclusion criteria were included in both a narrative synthesis and a quantitative meta-analysis.

**Results:**

Six studies met the inclusion criteria and were included. The results showed that the FFM trait extraversion demonstrated a small but positive association with alcohol consumption, and the trait conscientiousness showed a stable inverse relationship with alcohol consumption. In contrast, findings for the FFM traits agreeableness, openness to experience, and neuroticism were heterogeneous and did not reveal consistent patterns of association. Overall, the associations between FFM traits and alcohol consumption in adolescence were modest and generally weaker than those reported in adult populations and underscore the importance of adopting a developmental perspective when interpreting the results.

**Conclusion:**

This meta-analysis highlights contradictory findings regarding the associations between adolescent alcohol consumption and the FFM of personality traits. While extraversion and conscientiousness appear to be modestly related to alcohol use, evidence concerning agreeableness, openness, and neuroticism remains inconclusive. Accordingly, future research should investigate potential moderating factors, including peer influence and family environment, to further clarify how personality traits interact with contextual influences in the development of alcohol consumption during adolescence.

## Teaser

**Short summary:** This meta-analysis examined associations between adolescent alcohol consumption and Five-Factor Model traits. Extraversion showed a small positive association, whereas conscientiousness demonstrated a small inverse relationship. Other traits yielded inconsistent findings. Associations were modest and weaker than in adults, highlighting the importance of integrating developmental and contextual perspectives in future research.

## Introduction

Adolescence is a critical developmental period marked by significant biological, cognitive, and social changes (Steinberg 2014). During this stage, individuals start to form personality patterns that often persist into adulthood (Roberts and DelVecchio 2000). Early and heavy drinking in adolescence has been linked to enduring consequences, including heightened risks of alcohol misuse in adulthood (Hingson et al. 2006, McCambridge et al. 2011), increased likelihood of alcohol-related injuries in adulthood (Hingson and Zha 2009), as well as lasting damage to the frontal lobe (Crews and Boettiger 2009, Weiland et al. 2014).

Building on these findings, studies have investigated whether personality traits are linked to patterns of alcohol consumption in adulthood, with systematic reviews and meta-analyses consistently demonstrating associations between the Five-Factor Model (FFM) (McCrae and Costa Jr 2008) and alcohol-related outcomes in adult populations.

For instance, a systematic review by Adan et al. (2017) found that individuals who engage in binge drinking tend to exhibit higher neuroticism, higher extraversion, and lower conscientiousness. Similarly, a pooled analysis of 72 949 adults by Hakulinen et al. (2015) showed that higher extraversion and lower conscientiousness were significantly associated with greater alcohol consumption. A meta-analysis including 20 studies by Malouff et al. (2007) reported that low conscientiousness and agreeableness, along with high neuroticism, were linked to greater alcohol involvement. Lui et al. (2022) clarified these relationships, demonstrating that higher conscientiousness and agreeableness predicted lower alcohol consumption and fewer negative drinking-related consequences, whereas higher neuroticism and extraversion were associated with increased alcohol consumption and risky drinking behaviors. Despite these observations in the connection between FFM traits and alcohol related outcomes in adults, this relationship remains unclear for adolescents. There are reasons to believe that the relationship between personality and substance consumption may differ when studying the adult population and adolescents. For instance, Jones et al. (2022) found a significant time-variant association for conscientiousness, where lower conscientiousness was associated with higher alcohol consumption in early and late adolescents but not adults.

Conflicting results between adults and adolescents have also been demonstrated when studying FFM and smoking. For instance, Malouff et al. (2006) found that smoking was positively associated with neuroticism and negatively associated with conscientiousness and agreeableness, and showed no significant association with extraversion or openness to experience. In contrast, Zvolensky et al. (2015) did a 10-year longitudinal study between FFM traits and smoking (measured as lifetime cigarette use, progression to daily smoking, and persistent smoking) in adults. Here, results indicated that smoking was positively associated with neuroticism and openness to experience, and negatively associated with conscientiousness, while extraversion and agreeableness showed no clear relationship. Conner et al. (2009) showed that there was a positive association between neuroticism and smoking, a negative association between conscientiousness and smoking, and a weak positive association between extraversion and smoking. Yáñez et al. (2017) also found a positive association between neuroticism and smoking, a positive association between extraversion and smoking, and a negative association between conscientiousness and smoking.

Based on these studies, neuroticism was consistently positively associated with smoking, and conscientiousness was consistently negatively associated in both adults and adolescents. However, extraversion showed a positive association with smoking mainly in adolescents, while agreeableness and openness showed mixed or age-dependent associations. This suggests that the influence of personality traits on substance consumption may vary across developmental stages, indicating that the age-dependent patterns observed in smoking could also extend to the association between FFM traits and other forms of substance use, such as alcohol consumption. Given that alcohol and tobacco use are often positively correlated among adolescents (Grucza and Bierut 2006), it is plausible that the developmental variations identified in smoking behaviors may likewise apply to alcohol consumption.

Taken together, these observations suggest that the relationship between alcohol consumption and the FFM personality traits may differ across developmental stages, paralleling the variations observed in smoking behaviors (Malouff et al. 2006, Conner et al. 2009, Zvolensky et al. 2015, Yáñez et al. 2017). Accordingly, the present systematic review aims to identify if FFM personality traits are consistently associated with the frequency and volume of alcohol consumption among adolescents.

## Materials and methods

### Database search methodology

The study followed PRISMA guidelines (Page et al. 2021) for conducting a systematic review. It was pre-registered in PROSPERO (Registration ID: CRD420251025651). A systematic database search was conducted on the 18th of June 2025, using PsycINFO and PubMed. The following search terms were applied in both databases: tiab("big 5" OR “big five” OR "5 factor" OR "five factor") AND tiab(alcohol). The searches were age-restricted to “School Age (6–12),” “Adolescence (13–17 years),” and “Young Adulthood (18-29)” in PsycINFO and “Child (6–12),” “Adolescents (13–18),” and “Young adult (19–24)” in PubMed.

### Inclusion criteria

(a) Original peer-reviewed studies in English with quantitative data.
(b) Focused on adolescents aged 10–19, as per the definition of adolescents by the World Health Organization (2019).
(c) Variables of interest: Traits of the FFM (McCrae and Costa Jr 2008) and alcohol consumption of adolescents.
(d) Alcohol consumption should be measured as frequency of consumption or volume of alcohol consumed.
(e) Studies analyzing FFM traits or alcohol consumption as moderators were included, provided the specific association between variables was explicitly reported.
(f) Studies with data from broader age ranges were included if adolescent data (10–19) were analyzed separately.
(g) No preference for the instrument measuring FFM traits, but studies must include at least one of the five traits.

### Study selection

Four authors (A.P., G.E., S.A., and T.W.S.) independently screened the titles and abstracts using Rayyan (Ouzzani et al. 2016). All authors discussed any conflicts that arose and resolved them by reaching a consensus. Nineteen full-text articles were retrieved and screened for eligibility against the predetermined inclusion criteria.

### Data extraction

Data (study design, participants’ characteristics, and measured outcome results) were independently extracted by four authors (A.P., G.E., S.A., and T.W.S.) using Rayyan. Extracted study characteristics were sample size, sample characteristics (age, gender, nationality, and how many participants actively consume alcohol), study design, Big Five personality instrument, alcohol measure, limitations, statistical tool, and results.

### Measures

“Alcohol consumption” was specified as either frequency of alcohol consumption or quantity of alcohol consumed (e.g. standard consumption alcohol units). The outcome of the review measure is alcohol consumption. In the selected studies, alcohol intake was measured using self-report questionnaires or previously validated scales (The Alcohol Intake Scale [AIS]). Two studies (Ibáñez et al. 2015, Gallego et al. 2018) used the standard consumption alcohol units (SDU) measurements according to their country’s guidelines.

Personality was conceptualized through the FFM, comprising Extraversion, Agreeableness, Conscientiousness, Neuroticism, and Openness to Experience (Costa Jr and McCrae 1995, McCrae and Costa Jr 2008).

### Data synthesis and analysis

A random-effects meta-analysis model was fitted using the calculated effect sizes and the variances for each of the five Big 5 traits. To assess heterogeneity, we calculated *Q*-tests and *I*^2^ for each trait. Analyses were conducted using the metafor package (Viechtbauer 2010) for RStudio. The strength of associations was interpreted according to Cohen’s guidelines, with effect sizes of 0.10, 0.30, and 0.50 representing small, medium, and large effects, respectively (Cohen 1988).

### Bias assessment

As all included studies were based on cross-sectional data, the AXIS tool (Downes et al. 2016) was used to systematically appraise their risk of bias. The assessments were made by three of the authors (A.P., G.E., S.A.), where each article was assessed by two authors.

## Results

The systematic literature search generated 212 records (PsycINFO, *n* = 119, PubMed, *n* = 93). After removing 49 duplicates, 163 abstracts were screened, of which 144 were excluded for not meeting the inclusion criteria. Nineteen full-text articles were assessed, with exclusions due to differing populations (*n* = 6), lack of reporting relevant personality and alcohol outcomes (*n* = 5), or absence of alcohol frequency data (*n* = 2). Six studies met the inclusion criteria and were included (Merenäkk et al. 2003; Ibáñez et al. 2015; Salemink et al. 2015; Gallego et al. 2018; Geusens et al. 2019; Yañez et al. 2020) (see Appendix A for PRISMA flow chart).

### Participant and study characteristics

This review contains data from 3221 adolescents across six European studies (see Table 1). The mean age was 15.02 years (range = 12–17), with a relatively balanced gender distribution. The analyses were based on participants reporting alcohol consumption, which varied across studies from as low as 19.9% (Yañez et al. 2020) to as high as 78% (Merenäkk et al. 2003). All studies employed a non-experimental cross-sectional design, though measurement instruments differed (see Table 1).

**Table 1:** Description of included studies and participants' characteristics.

Included articles	*N*	Age	Gender_1_	% of consum ers of alcohol	Nationality	Study design	Measure for alcohol consumption	Big 5 measurement
Gallego et al. (2018)	120 221	12–15 years, *M*_age_ = 13.68, 12–15 years, *M*_age_ = 12.53	52% female 55% female	NA NA	Scottish Spanish	Correlation study	AIS (Ibáñez et al. 2015), ^a^Frequency of alcohol consumption_2_ and ^a^SDUs per weekend_3_	JS NEO_9_ NEO–PI–3
Geusens et al. (2019)	866	14-16 years *M*_age_ = 14.85 (SD = .71)	57.7% female	52.3%	Belgian		^a^Frequency of alcohol consumption_4_, and ^a^Amount consumed on typical day of drinking_5_	44-item Big Five Inventory (John and Srivastava 1999)
Ibáñez et al. (2015)	361	14–16 years *M*_age_ = 15.16 (SD = .60)	58.7% female	56%	Spanish	Correlation study and, regression study	AIS-UJI_6_	JS NEO_9_
Merenäkk et al. (2003)	591	14–17 years *M*_age_ = 15.4 (SD = .60)	56% female	78%	Estonia	Cross sectional	^a^Frequency of alcohol consumption_7_	^a^44 item measure_10_
Salemink et al. (2015)	21 9^13^	*M*_age_ = 17.0 (SD = .60)	49% female	100%	Dutch	Longitudinal correlation study	^a^Frequency of alcohol consumption_11_	30 item measure_12_
	24 5^13^	*M*_age_ = 17.0 (SD = .50)	46% female	100%	Dutch	Longitudinal correlation study	^a^Frequency of alcohol consumption_11_	30 item measure_12_
Yañez et al. (2020)	695	14–15 years *M*_age_ = 14.5 (SD = .66)	57% female	19.9%	Spanish	Between subject design	^a^Frequency of alcohol consumption_8_	BFQ-CA

a, scale created by author. 1 = remainder of participants are male, 2 = measured on a 4-point scale (1 = Never or almost never, 2 = 1–3 times per month, 3 = 1–6 times per week, 4 =  daily), 3 = As the definition of an SDUdiffer between Spain and the UK, they were instead converted into grams of alcohol for analysis (8g, 10g), 4 = measured on a 5-point scale (0 = never to 4 = 4 times per week or more), 5 = measured on scale (0 = nothing to 5 = 10 glasses or more), 6 = measures the typical amount of consume alcohol consumed during weekdays and weekends, which is then converted into SDUs (10grams), 7 = asked on a 5-point scale (1 = never to 5 = everyday), participants were categorized into three groups: non-consumers (including those who had not tasted alcohol and those who had tasted but reported that they never consumed it), moderate consumers (who consumed alcohol less often than once per week), and frequent consumers (once per week or more often), 8 = Measured on 4-point scale(1 = never-almost never; 2 = sometimes a month; 3 = on weekends; 4 = daily or almost daily), 9 = Spanish adaptation of NEO-PI-R, 10 = based on the CCQ-set (John, et al. 1994), the questionnaire was filled out by the participants mothers, 11 = At T1: Asked whether they had ever drunk alcohol, how often they had drunk during the past four weeks on a 6-point Likert scale, and how often they had drunk more than 6 glasses of alochol during the past four weeks on the same 6-point Likert scale. At T2: Asked how often they had drunk alcohol during the past four weeks on a 6-point Likert scale, 12 = Shortened Dutch version of Goldberg’s Big Five Questionnaire, by Meeus et al. (2011), 13 = The two samples overlap with 97 participants. Scotland (UK), Spain, Estonia, and the Netherlands have a legal drinking age of 18 years, while Belgium allows beer/wine at 16 years and spirits at 18 years.

#### Measurements

As depicted in Table 1, personality was assessed using a range of Big Five instruments. The JS-NEO was employed by Ibáñez et al. (2015) and Gallego et al. (2018), the latter of whom also used the NEO-PI-3 for their Scottish sample. Merenäkk et al. (2003) used the California Child Q-set (CCQ), completed by parents, whereas Yañez et al. (2020) used the BFQ-CA. Geusens et al. (2019) used a 44-item Big Five measure (John and Srivastava 1999), and Salemink et al. (2015) employed a shortened Dutch version of Goldberg’s Big Five Questionnaire.

Alcohol consumption was operationalized heterogenously across studies. Five assessed consumption frequency (Merenäkk et al. 2003; Salemink et al. 2015; Gallego et al. 2018; Geusens et al. 2019; Yañez et al. 2020), while several additionally measured intake using SDUs either as amount consumed on a typical day of drinking (Geusens et al. 2019) or across the week/weekend (Ibáñez et al. 2015; Salemink et al. 2015; Gallego et al. 2018).

#### Narrative review

All articles except Gallego et al. (2018) found significant results between the extraversion trait and alcohol usage (see Table 2). Four articles (Merenäkk et al. 2003; Salemink et al. 2015; Ibáñez et al. 2015; Geusens et al. 2019) found significant weak positive correlations, whereas Yañez et al. (2020) reported that adolescents high in extraversion were nearly twice as likely to consume alcohol weekly as those with low extraversion scores.

**Table 2:** Data extractions of results.

Included articles		FFM traits					Traits correlated with	Analysis
		E	O	C	A	N		
Gallego et al. (2018)_a_	*r (scot)*	.14	−.06	−.17	−.26^**^	−.05	*1*	Correlation analysis
	*r (Spanish)*	0.09	−1.0	−.25^***^	−.32^***^	.04	*2*	
Geusens et al. (2019)	*r*	.16^***^	.02	−.16^***^	-	−.03	*4*	Correlation analysis
	*β*	.10^***^	.03	−.10^***^	-	.003	*4*	Linear regression analysis
Ibáñez et al. (2015)	*β*	.10^*^	−.12^*^	−.10^*^	-	-	*5*	Linear regression analysis
	*β*	-	-	-	−.14^*^	-	*6*	
Merenäkk et al. (2003)	*ρ*	.15^**^	.11^*^	−.08	−.01	−.02	*3*	Spearman’s bivariate correlation
Salemink et al. (2015)	*r*	.18^*^	-	-	-	−.18^*^	*9*	Pearson correlation
	*r*	.24^***^	-	-	-	−.18^*^	*10*	Pearson correlation
Yañez et al. (2020)	aOR	1.9^*^	0.7^*^	0.6^*^	1.0	0.7^*^8	*7*	Logistic regression analysis

^*^ *P* < .05, ^**^P < .01, ^***^P < .001. E = extraversion, O = openness to experience, C = conscientiousness, A = agreeableness, N = neuroticism. 1 = Alcohol frequency, 2 = Alcohol quantity on weekend, 3 = Alcohol consumption, frequency of most consumed type of alcohol, 4 = Alcohol consumption score, calculated by multiplying the typical amount consumed when drinking with the frequency of drinking per week, 5 = Weekend SDUs, 6 = Weekday SDUs, 7 = Consuming or not consuming alcohol weekly, − = No results reported, 8 = originally measured as “emotional stability,” and was thus reversed, 9 = Frequency of alcohol consumption at T1, 10 = Frequency of alcohol consumption at T2, 8 months later.

Findings regarding openness to experience were mixed. Approximately half of the studies reported non-significant associations, while those observing significant effects found only weak associations with alcohol consumption (Merenäkk et al. 2003; Gallego et al. 2018; Yañez et al. 2020). The direction of these associations also varied across studies. Ibáñez et al. (2015) and Yañez et al. (2020) found a negative association, whereas Merenäkk et al. (2003) noted a positive correlation between these variables. It should be noted that Salemink et al. (2015) did not include or report any data on this trait.

In terms of conscientiousness, all studies except Merenäkk et al. (2003) reported a significant negative correlation with alcohol consumption, although effect sizes were generally small. Salemink et al. (2015) did not include conscientiousness in their analyses.

Regarding agreeableness, two studies reported no significant association with alcohol consumption (Merenäkk et al. 2003; Yañez et al. 2020), whereas two studies found significant negative associations of small to moderate magnitudes (Ibáñez et al. 2015, Gallego et al. 2018). Agreeableness was not examined by Geusens et al. (2019) or Salemink et al. (2015); in the former, the variable was excluded due to poor internal consistency (Cronbach’s α = .63).

For neuroticism, studies reported no significant association with alcohol consumption. Exceptions included Yañez et al. (2020), who found that adolescents with higher neuroticism scores had a 30% lower likelihood of weekly alcohol consumption, and Salemink et al. (2015), who observed a weak negative correlation.

#### Statistical analysis

The meta-analysis was restricted to studies reporting correlation coefficients, resulting in the inclusion of four papers (Merenäkk et al. 2003; Salemink et al. 2015; Gallego et al. 2018; Geusens et al. 2019), which encompassed six independent experiments. Not all experiments reported data for every personality trait, resulting in different numbers of studies per trait.

Openness to experience was examined using three experiments (Merenäkk et al. 2003; Gallego et al. 2018; Geusens et al. 2019). Moderate heterogeneity was observed (τ^2^ = 0.003, *I*^2^ = 54%), though the heterogeneity test was not significant (*Q*(2) = 4.34, *P* = .11). The 95% prediction interval [−0.087 to 0.174] was narrow, suggesting that heterogeneity might not matter as the actual range of effect sizes was small. The pooled effect size was small and non-significant (*r* = 0.043, SE = 0.042, 95% CI [−0.038, 0.125], *P* = .30), indicating no robust evidence for an association, with any true effect likely to be very small (*r* < 0.12).

Conscientiousness was assessed using three experiments (Merenäkk et al. 2003; Gallego et al. 2018; Geusens et al. 2019). Moderate heterogeneity was found (τ^2^ = 0.0024, *I*^2^ = 48.1%), though not statistically significant (*Q*(3) = 5.44, *P* = .14). The 95% prediction interval [−0.0047, 0.3040] suggests that the true effect in a future sample could range from negligible to small-moderate. The pooled effect size was small and negative (*r* = −0.154, SE = 0.036, 95% CI [−0.226, −0.083], *P* < .001), suggesting a reliable negative association with alcohol consumption.

Extraversion was examined across six experiments, and no evidence of heterogeneity was found (τ^2^ = 0, *I*^2^ = 0%, *Q*(5) = 2.99, *P* = .70) (Merenäkk et al. 2003; Salemink et al. 2015; Gallego et al. 2018; Geusens et al. 2019). This indicated a small but significant positive association with alcohol consumption (*r* = 0.162, 95% CI [0.120, 0.200], *P* < .001), with a 95% prediction interval [0.12, 0.20] suggesting future studies are likely to find a small positive effect within a similar range.

Agreeableness was assessed using three experiments and demonstrated substantial heterogeneity (τ^2^ = 0.082, *I*^2^ = 95%, *Q*(2) = 30.97, *P* < .001) (Gallego et al. 2018; Merenäkk et al. 2003). The wide 95% prediction interval [− 0.63, 0.68] suggests considerable uncertainty regarding the true effect. The pooled effect size was negligible and non-significant (*r* = 0.022, 95% CI [−0.312, 0.356], *P* = .90), indicating no consistent association.

Neuroticism was investigated using six experiments, with moderate heterogeneity observed (τ^2^ = 0.0039, *I*^2^ = 57%, *Q*(5) = 10.60, *P* = .06) (Merenäkk et al. 2003; Salemink et al. 2015; Gallego et al. 2018; Geusens et al. 2019). The 95% prediction interval [−0.205, 0.075] suggests the true effect could range from a small negative to a negligible positive association. The pooled effect was marginally non-significant and small (*r* = −0.065, 95% CI [−0.133, 0.004], *P* = .064), indicating that if a true effect exists, it is likely small and negative (*r* < −0.13).

Overall, the meta-analytic findings largely support the narrative review regarding direction and magnitude of the associations, except for agreeableness. Forest plots for each trait are presented in Fig. 1 (see Appendix C for funnel plots).

**Figure 1:**
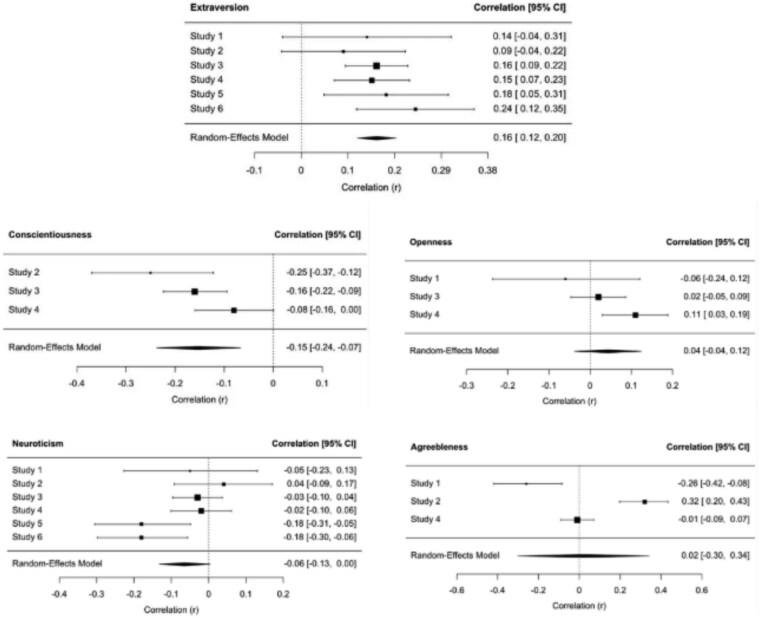
Forest plots displaying the pooled effects for Big Five traits.

### Bias assessment

All included studies relied on self-reported alcohol consumption, which may introduce recall bias. As shown in Table 2, Ibáñez et al. (2015) did not report all outcomes, resulting in outcome reporting bias, and conclusions could not be drawn for characteristics with missing data. Confounding bias may also be present, as Ibáñez et al. (2015) and Yañez et al. (2020) used regression analyses adjusting for other variables. Additional potential sources of bias include different measurements of alcohol consumption and personality, as well as differences between countries and cultures. Overall, the studies were considered to have low-to-moderate risk of bias, as assessed using the AXIS tool (see Appendix B for AXIS assessment).

Some data were unavailable for the meta-analysis, potentially introducing bias related to selective reporting. Specifically, Geusens et al. (2019) and Yañez et al. (2020) did not report the necessary bivariate estimates, and Ibáñez et al. (2015) only reported partial correlations. Despite attempts to obtain the original data, these studies could not be included. Consequently, the meta-analytic findings should be interpreted with caution, given the potential influence of missing data.

## Discussion

The findings from the narrative review and meta-analysis indicate that associations between personality traits and adolescent alcohol consumption are generally weak to modest and vary in consistency across studies. Extraversion emerged as the most consistently reported trait, showing a small but positive relationship with alcohol consumption in the narrative review and meta-analysis. Conscientiousness also demonstrated a stable pattern, with both the narrative review and meta-analysis identifying a small negative association with alcohol consumption across most studies. Openness to experience showed the most inconsistent results, with weak, non-significant, and highly variable associations in both direction and magnitude across samples, as reflected in the meta-analytic findings. Findings for agreeableness were mixed: the narrative review suggested weak negative or null associations, whereas the meta-analysis indicated highly heterogeneous and non-significant effects. Finally, neuroticism was largely unrelated to adolescent alcohol consumption, with both the narrative and meta-analytic findings pointing to small, non-significant associations, despite occasional reports of weak negative relationships. Overall, the convergence of narrative and quantitative evidence suggests that personality traits play only a limited role in explaining adolescent alcohol consumption.

When connecting this paper’s results to previous literature, we find that the findings on extraversion align with major meta-analyses conducted in adult populations (Hakulinen et al. 2015, Lui et al. 2022). However, they also contradict Malouff et al. (2007) (conducted in adults), where they found no significant association between extraversion and alcohol involvement. The present results for conscientiousness align with prior research in adult populations (Hakulinen et al. 2015; Adan et al. 2017; Lui et al. 2022), further establishing the trait association with alcohol consumption across age groups. However, the results for agreeableness remain unclear. Hence, there may be a difference between adults and adolescents regarding the relationship between agreeableness and alcohol consumption, but it cannot be concluded definitively. The findings on neuroticism align with some previous studies (Adan et al. 2017; Lui et al. 2022) but contradict others (Malouff et al. 2007, Hakulinen et al. 2015). Lastly, the openness to experience results are also in line with previous research, which reports contradictory findings (Hakulinen et al. 2015; Adan et al. 2017; Lui et al. 2022). Hence, the uncertainty of the role of openness in alcohol consumption is still present in the context of adolescents.

In sum, the present findings suggest a weaker association between FFM traits and alcohol consumption in adolescents compared to the more robust relationship observed in adults. This discrepancy is noteworthy because adolescence is characterized by heightened impulsivity and risk-taking (White and Hingson 2019), which are considered developmentally normative and do not necessarily reflect a stable personality trait. For many young people, this increased propensity for risk-taking is temporary and specific to this developmental period, which may limit the predictive utility of FFM traits for alcohol consumption during adolescence.

Developmental factors may explain these weaker associations in adolescents. Although the FFM conceptualizes personality traits as relatively enduring characteristics, research indicates that personality continues to develop into early adulthood (Van Dijk et al. 2020). During this stage, adolescents undergo major neurobiological, cognitive, and social development, which may temporarily weaken the strength of the association between personality traits and specific behaviors such as alcohol consumption. In adulthood, however, the traits have generally become more consistent across time (Roberts and DelVecchio 2000). This, in turn, could make adults’ behaviors, such as alcohol consumption, more stable, and trait-dependent. This developmental perspective may therefore explain why associations between personality traits and alcohol consumption tend to appear stronger and more reliable in adult samples compared to adolescent samples.

Another factor that may further influence these patterns is the environment and context. These factors likely operate through different mechanisms, and distinguishing among them may help explain why their associations with alcohol consumption vary. Extraverted adolescents tend to participate in larger peer networks and might therefore be more frequently exposed to opportunities for drinking (Gallego et al. 2018). In contrast, conscientious adolescents exhibit a lower likelihood and frequency of alcohol consumption even after accounting for parental monitoring and peer exposure, suggesting that self-regulatory traits may serve as a protective factor against early drinking (Hagger-Johnson et al. 2012). Adolescents who experience greater parental support are also less likely to engage in alcohol consumption (LaFreniere et al. 2021). Importantly, susceptibility to peer influence is particularly pronounced during adolescence, rendering peer-related environmental pathways especially salient during this developmental period (Steinberg and Monahan 2007). Similarly, parental support and monitoring exert a stronger influence on psychosocial adjustment in adolescence than in adulthood, highlighting the heightened impact of external influences during this period (Lansford et al. 2018). As these external constraints diminish in adulthood, personality traits may translate more directly into behavior.

Generational shifts may also contribute to the observed inconsistencies. Over the past two decades, adolescents have demonstrated significant changes regarding both alcohol consumption and personality traits (Smits et al. 2011, Raninen 2020). In Sweden, for instance, the proportion of 15–16-year-old who consume alcohol has halved since the early 2000s, with those who drink doing so less frequently and in smaller quantities (Raninen 2020). Research also suggests that more recent cohorts of younger people score slightly higher on conscientiousness and lower on traits associated with emotional instability, indicating greater self-control than earlier cohorts (Smits et al. 2011). Such cohort differences in personality may partly explain the noted decline in alcohol consumption among young people (Smits et al. 2011).

Overall, given the current inconsistencies and limited clarity in the literature, universal prevention strategies remain essential and should be implemented for all adolescents until a clearer understanding of the potential association between personality traits and alcohol consumption is established.

### Limitations and strengths

This systematic review and meta-analysis are not without limitations. A first limitation, affecting both the systematic and the meta-analytic findings, concerns the lack of standardization in the operationalization of “alcohol consumption” across the included studies. Although all of the included studies investigated alcohol consumption in adolescent samples, the specific definitions and measurement strategies varied considerably. For instance, some studies used simple categorical definitions (e.g. weekly versus non-weekly consumption; Yañez et al. 2020), while others used complex composite indices combining frequency and quantity (e.g. Geusens et al. 2019). This variability may introduce conceptual heterogeneity into both the findings of the systematic review and the effect sizes derived from the meta-analysis, as the included measures may not capture a single underlying construct but rather related yet distinct dimensions of alcohol consumption. Consequently, the pooled estimates should be interpreted with caution, as they may reflect methodological heterogeneity that could either attenuate or inflate the true associations between personality traits and alcohol consumption in adolescents. Future research would therefore benefit from using standardized, validated measures of alcohol consumption to improve comparability across studies and yield more precise estimates in subsequent meta-analyses on this topic.

Variations in sample drinking patterns across studies may also have affected the results of this review. Because some samples consisted of a higher number of drinkers than others, the strength of the relationships reported by each study cannot be interpreted in the same way. Importantly, the association between personality traits and alcohol consumption may operate in different directions depending on the distribution of drinking behavior within the sample. If personality traits are primarily related to whether adolescents drink at all, the association may, in some cases, be stronger in samples with a lower proportion of drinkers. For example, if extraversion is mainly associated with the transition from non-drinking to drinking, this pattern may be more visible in samples in which a smaller proportion of adolescents report alcohol use. By contrast, in samples where a large majority already report drinking, there may be less variation in drinking initiation, which could make such associations more difficult to detect. At the same time, in these samples, personality traits may instead relate to variation in frequency or quantity of consumption. It is therefore important to consider both possibilities when interpreting differences in effect sizes across studies.

The low mean age of 15.02 years for all included participants in this review should also be considered, as the results may not apply to older adolescents.

The findings should further be interpreted with caution, given the limited number of included studies. In particular, several meta-analytic estimates were based on as few as three studies per personality trait, which reduces the robustness and precision of the results and limits the reliability of conclusions regarding the associations.

Reliance on self-reported measures of alcohol consumption may introduce recall bias, as adolescents may inaccurately estimate their drinking behavior due to memory limitations or social desirability. In addition, variability in outcome reporting across studies complicates direct comparisons and could introduce selective reporting bias. Together, these factors may affect the accuracy and consistency of the data and limit the robustness of the overall bias assessment.

An additional limitation is that all included studies were conducted in European countries. Cultural norms, legal drinking ages, alcohol availability, and social attitudes toward adolescent drinking vary substantially across regions, which may influence both alcohol consumption patterns and the expression or behavioral relevance of personality traits. Consequently, the findings may not generalize to non-European populations, and cultural bias cannot be ruled out. Future research should include more regional and culturally diverse samples to determine the cross-cultural robustness of the association between personality traits and alcohol consumption during adolescence.

Although the observed heterogeneity in the results of the statistical analysis would warrant an analysis of moderating variables, conducting a meta-regression was not appropriate due to the small number of included studies (3–6 per personality trait), which is well below recommended thresholds (Deeks et al. 2024). Additionally, potential moderators were not suitable for analysis. For instance, age was reported in broad, inconsistent ranges without detailed distributions (e.g. 12–15 years or 14–16 years), and cultural context showed limited variability, as all studies were conducted in similar European settings. Consequently, any meta-regression would likely yield unreliable or uninformative results.

It has previously been demonstrated that alcohol-related outcomes shift significantly during different developmental periods within adolescence. However, this paper is unable to acknowledge that such patterns (in both personality and alcohol use) can vary considerably across early, mid-, and late-adolescence (Hagborg et al. 2020). Given that most included studies primarily involved younger adolescents, the developmental range of the samples was limited, which restricts the ability to draw conclusions about how associations between personality and alcohol consumption may differ across the full span of adolescence, particularly among older adolescents.

## Conclusion

This meta-analysis highlights the contradictory results on the associations between adolescent alcohol consumption and the FFM of personality traits. While extraversion and conscientiousness appear to be modestly related to alcohol use, evidence concerning agreeableness, openness, and neuroticism remains inconclusive. Given these inconsistencies, developing FFM-based prevention and intervention programs for adolescents would be premature.

## Supplementary Material

R2_Manuscript_ID_ALC-26-0036-Appendix_agag048

R2_Manuscript_ID_ALC-26-0036-Appendix_1_agag048

R2_Manuscript_ID_ALC-26-0036-Appendix_3_agag048

## Data Availability

All data analyzed in this study are derived from previously published studies, and data sharing is therefore not applicable.
