# Supplementary material for: Linking five-factor personality model to alcohol consumption in adolescence: a systematic review and meta-analysis
Source: Alcohol Alcohol. 2026 Aug 10;61(5):agag048. doi: 10.1093/alcalc/agag048 (PMC13629348; doi:10.1093/alcalc/agag048)
Supplement: Supplementary file 1 [file r2_manuscript_id_alc-26-0036-appendix_agag048.docx]

## Appendix A

### PRISMA

## Appendix B

### AXIS assessment of bias

|  | Gallego et al. (2018) | Geusens et al. (2019) | Ibáñez et al. (2015) | Merenäkk et al. (2003) | Yañez et al. (2020) | Salemink et al. (2015) |
| --- | --- | --- | --- | --- | --- | --- |
| 1: Were the aims/objectives of the study clear? | yes | no | yes | yes | yes | yes |
| 2: Was the study design appropriate for the stated aim(s)? | yes | yes | yes | yes | yes | yes |
| 3: Was the sample size justified? | no^1^ | no^1^ | no^1^ | no | no | no |
| 4: Was the target/reference population clearly defined? (Is it clear who the research was about?) | yes | yes | yes | yes | yes | yes |
| 5: Was the sample frame taken from an appropriate population base so that it closely represented the target/reference population under investigation? | yes | yes | yes | yes | yes | yes |
| 6: Was the selection process likely to select subjects/participants that were representative of the target/reference population under investigation? | yes | yes | yes | yes | yes | yes |
| 7: Were measures undertaken to address and categorise non-responders? | no | no | no | yes | no | no |
| 8: Were the risk factor and outcome variables measured appropriate to the aims of the study? | yes | yes | yes | yes | yes | yes |
| 9: Were the risk factor and outcome variables measured correctly using instruments/measurements that had been trialled, piloted or published | yes | no^2^ | yes | yes | yes | yes |
| 10: Is it clear what was used to determine statistical significance and/or precision estimates? (eg, p values, CIs) | yes | no | yes | yes | yes | yes |
| 11: Were the methods (including statistical methods) sufficiently described to enable them to be repeated? | yes | no | yes | no | yes | no |
| 12: Were the basic data adequately described? | yes | no | yes | yes | yes | no |

|  | Gallego et al. (2018) | Geusens et al. (2019) | Ibáñez et al. (2015) | Merenäkk et al. (2003) | Yañez et al. (2020) | Salemink et al. (2015) |
| --- | --- | --- | --- | --- | --- | --- |
| *13: Does the response rate raise concerns about non-response bias? | no | no | no | no | no | unsure |
| 14: If appropriate, was information about non-responders described? | no | no | no | yes | no | no |
| 15: Were the results internally consistent? | yes | yes | yes | yes | yes | no |
| 16: Were the results for the analyses described in the methods, presented? | yes | yes | yes | yes | yes | yes |
| 17: Were the authors’ discussions and conclusions justified by the results? | yes | yes | yes | yes | yes | yes |
| 18: Were the limitations of the study discussed? | yes | yes | yes | yes | yes | yes |
| *19: Were there any funding sources or conflicts of interest that may affect the authors’ interpretation of the results? | unsure | no | no | no | no | no |
| 20: Was ethical approval or consent of participants attained? | yes | no | yes | yes | yes | yes |
| Reviewer initials | S.A., G.E. | S.A, G.E. | S.A., A.P. | S.A., A.P. | A.P., G.E. | A.P., G.E. |
| Total Score (Based on Yes Responses) | 16 | 11 | 17 | 18 | 17 | 13 |
| Total % | 80% | 55% | 85% | 90% | 85% | 65% |
| Risk Level | Low | Moderate | Low | Low | Low | Moderate |

*Note*: *no is interpreted as yes in scoring, 1 = Sample size was not justified, 2 = Aside from the FFM scale no other instrument had been trialled/published/piloted before. Risk Levels- “Low” if the majority (≥70%) of the items were answered as “Yes”; “Moderate” if 50%–69% of items were answered as “Yes”, and “High” if < 50% were answered as “Yes”.

#### Appendix C

### Funnel plots
